# An Optimized MicroPET Imaging Method for the Distribution and Synergies of Natural Products

**DOI:** 10.3389/fphar.2018.00948

**Published:** 2018-08-21

**Authors:** Qingxin Cui, Yang Liu, Mengge Zhou, Yanqi Han, Chengcheng Yin, Gang Bai

**Affiliations:** College of Pharmacy, State Key Laboratory of Medicinal Chemical Biology, Tianjin Key Laboratory of Molecular Drug Research, Nankai University, Tianjin, China

**Keywords:** [^18^F]-glycyrrhetic acid, platycodon grandifloras polysaccharides, dosimetry, biodistribution, positron emission tomography

## Abstract

**Purpose:** Understanding the distribution and interaction of the Traditional Chinese Medicines (TCMs) is an integral source of herbal drug discovery. An optimized radio-labeled method was explored that could conduct *in situ* biodistribution studies in animals. We evaluated the feasibility of the method and applied glycyrrhetinic acid and platycodon (PG) polysaccharides as models.

**Procedures:** [^18^F]-GA is a novel radiotracer which was performed positron emission tomography (PET) studies to assay the biodistribution of GA in mice. In addition, PG polysaccharide was used to intervene the biodistribution and dosimetry of GA. Scanning data were analyzed with professional software.

**Results:** Record the time-activity curves for all organs then use the normalization method to calculate the area under the curve as a dosimetry for each organ. Moreover, the addition of PG polysaccharides can significantly improve the dosimetry of GA in the lungs, and its effect was related to the administration time.

**Conclusion:** MicroPET imaging opens up a new avenue for the application of drug interactions between the TCMs.

## Introduction

Traditional Chinese Medicine (TCM) has many compounds with complex structures and multiple biological activities (Amirkia and Heinrich, 2015). Therefore, it has become very difficult to find a target organ or tissue of a certain compound. This needs a novel strategy of biodistribution research on the basis of bioactive compounds involved in the important biological phenomena and processes (Zhang et al., 2011; Futamura et al., 2013). However, it is widely recognized that tissue distribution of natural products is a huge task, although many successful examples of molecules target confirmation have been reported, such as blood concentration monitoring, liquid chromatography-mass spectrometry (LC-MS), and so on (Takahashi et al., 2010). These technology were conventional methods for drug tissue distribution (Dixon et al., 2007); however, the most serious issue preventing such an approach is the difficulty in a large number of animals and repeated experiments, time-consuming and laborious. More importantly, there are individual differences among animals inevitably, the systematic error between isolated organ and living animals will influence the results of the experiment (Phelps, 2000). So it is needed to seek new tools and methods for *in vivo* experiments. Positron emission tomography (PET) has provided a new testing tool and method for the study of the imaging and pharmacokinetics of small animal *in vivo* (Matsumura et al., 2003). PET can image molecules of biological processes and provide quantitative information sensitively. This can detect biological abnormalities in nervous system diseases even before symptoms appear (Peng and Levin, 2010). This classic anatomical approach is easily applied to many facilities at a lower cost. With the advancement of whole-body imaging equipment, PET imaging of non-human primates is preferred in several well-equipped research institutes because of the differences between species (Jiang et al., 2018). Therefore, radiation dosimetry assay using whole body PET in mice has been shown to be very helpful in the diagnosis of several clinical diseases (Sakata et al., 2013).

To demonstrate this approach, we chose to study the biodistribution of several biologically active natural products in the world. Among them, licorice showed its special advantage and supported its feasibility as an example to examine the whole-body PET method. Since licorice can produce glycyrrhetic acid (GA) under the hydrolysis of enzyme in the human body, GA is essentially the same as licorice in the pharmacological action (Li et al., 2014). The anti-inflammatory activity of GA was significantly affirmed and GA was used as an herbal medicine in the Mediter-ranean regions and in certain parts of Asia, it has been proven to have multiple biological activities (Zhao et al., 2017). GA exerted its anti-inflammatory activity by inducing an antioxidant defense system, reducing lipid peroxidation, and improving histological and oxidative ingery (Yang et al., 2017). It can also greatly reduce the production of excessive inflammatory factors, inhibit mRNA levels of active enzyme, and inhabit LPS-induced expression of TNF-a and IL-6 in quantitative dependence (Wang et al., 2011; Ishida et al., 2013).

Platycodon grandifloras (PG) is widely cultivated in East Asia and Eastern Siberia, as an ornamental plant and traditional herb medicine, has been used as a health food and medicine for the treatment of cough, chest tightness, bronchitis, pleurisy, etc. (Zhang et al., 2015; Zheng et al., 2017). The research on PG mainly focused on its biological activities, including its anti-tumor, anti-oxidation, and immune-enhancing effects. PG polysaccharides have immunostimulatory activity (Zheng et al., 2017), induce DC maturation and increase the expression levels of MHC-I/II, co-stimulatory molecules and cytokines, which can activate T cells (Park et al., 2014; Zheng et al., 2017). However, the effects of PG polysaccharides on the distribution of GA have remained elusive.

In this paper, we assayed the biodistribution of [^18^F]-GA in organs of mice *in vivo* using MicroPET imaging. The retention time of GA in mouse organs was calculated. It was demonstrated that was consistent with the literature reported. Then we detected the concentrations of GA in each organ by the intervention of PG polysaccharides in real time. In addition, different administration ways of PG polysaccharides were evaluated. The results demonstrated that taking GA and PG polysaccharides at the same time could significantly change the distribution of GA, and the lung became the main target organ.

## Materials and Methods

### Reagents and Chemicals

Glycyrrhetic acid was purchased from Sigma Corporation (St. Louis, MO, United States). 1-Ethyl-3-(3-dimethylaminopropyl) carbodiimide (EDC), P-toluenesulfonyl chloride and N-hydroxysuccinimide (NHS) were purchased from Alfa Aesar (Haverhill, MA, United States). Kryptofix2.2.2 (K2.2.2) was purchased from ABX Corporation (Radeberg, Germany). H_2_^18^O was purchased from Shanghai Research Institute of chemical industry. Sep-Pak C_18_ column was purchased from Waters (United States). Dichloromethane (CH_2_Cl_2_), ethyl acetate (EtOAc), acetonitrile (MeCN), and methanol (MeOH) were purchased from Phytomarker Co., Ltd. (Tianjin, China). PG polysaccharides were purchased from Changan Chinese Herbal Medicine Co., Ltd. (Hebei, China). Double distilled water was obtained from the Millipore filtering system (Tokyo, Japan). All the chemicals and solvents used in the paper were analytical grade.

### Synthesis of [^18^F]-Glycyrrhetinic Acid

**SCHEME 1:**
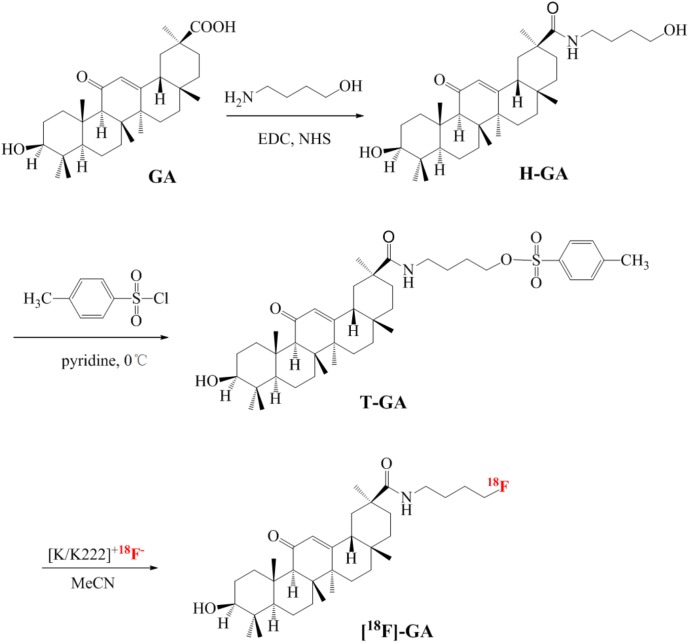
The synthetic strategy of [^18^F]-GA.

The general strategy for synthesis of [^18^F]-Glycyrrhetinic acid is shown in **Scheme 1**. To a stirred solution of Glycyrrhetinic acid (0.950 g, 2 mmol, 1 Eq) in CH_2_Cl_2_, EDC (0.950 g, 5 mmol, 2.5 Eq) was added, and the mixture stirred below freezing point for 30 min. Then 1 mL 4-amino-1-butanol (16 mmol, 8 Eq) was added to the mixture. The reaction mixture was diluted with CH_2_Cl_2_ and washed with 1 N aq HCl, 5% aq NaHCO_3_, and then brine. The organic layer was gathered and dried by anhydrous Na_2_SO_4_, filtered, and concentrated under reduced pressure to give the Hydroxylation-Glycyrrhetinic acid (H-GA) (0.739 g, 47%) as white solid (Lassalas et al., 2016). *R*f = 0.5 (EtOAc/MeOH 1:1); mp 295°C; ^1^H NMR [CH_3_OD, 400 MHz] δ8.02–7.97(m, 1H), 5.62–5.57(m, 1H), 3.65(d, 2H), 3.46(d, 2H), 3.15(m, 1H), 2.79–2.74(m, 1H), 2.44(s, 3H), 1.45–0.80(m, 42H). ^13^C NMR [CH_3_OD, 100 MHz] δ36.935, 37.343, 38.829, 38.947, 41.236, 41.447, 43.193, 43.443, 45.297, 54.811, 60.332, 61.745, 78.019, 127.712, 171.174, 177.715, 201.208. HRMS (EI) for C_34_H_55_NO_4_ (M^+^) calcd 541.4109, found 541.4111.

To a stirred solution of H-GA (0.260 g, 0.5 mmol, 1 Eq) in anhydrous pyridine, P-toluenesulfonyl chloride (0.100 g, 0.5 mmol, 1 Eq) was added, and the mixture stirred below freezing point for 6 h. Using silica gel column chromatography for separation, thin layer chromatography (TLC) for tracking and detection and the Tosylation-Glycyrrhetinic acid (T-GA) was obtained as white solid. Rf = 0.4 (PE/EtOAc1:1); mp 298°C; ^1^H NMR [CDCl_3_, 400 MHz] δ7.75 (d, 2H), 7.27(d, 2H), 5.56–5.60(m, 1H), 4.21(d, 2H), 3.76(d, 2H), 3.15(m, 1H), 2.79–2.74(m, 1H), 2.44(s, 3H), 1.45–0.80(m, 42H). ^13^C NMR [CDCl_3_, 100 MHz] δ15.328, 15.374, 16.541, 17.626, 20.604, 22.348, 23.532, 25.534, 26.960, 27.554, 28.078, 30.599, 30.871, 31.620, 35.701, 36.160, 37.116, 37.875, 40.627, 42.222, 44.313, 46.956, 53.610, 54.318, 60.554, 66.054, 89.685, 126.580, 127.286, 128.650, 134.111, 143.250, 168.771, 170.548, 198.750. HRMS (EI) for C_41_H_61_NO_6_S (M^+^) calcd 695.9938, found 695.9937.

[^18^F] fluoride ions obtained from proton-irradiated ^18^O-water were trapped in a QMA cartridge (Sep-Pak Light QMA cartridge, Waters) and then eluted from the column using a solution of K_2_CO_3_ (7 μmol), Kryptofix 2.2.2 (130 μmol) in water and MeCN (Constantinescu et al., 2013b). The solution was evaporated at 116°C under a stream of nitrogen to leave a yellow residue (Jiang et al., 2018). Then T-GA (7 mg, 0.01 mmol) in MeCN (1 mL) was added in the residue when cooled to room temperature. The reaction bottle was quickly heated to 90°C for 5 min, after which the reaction solution was distilled under a stream of nitrogen (50 mL/min) for 5 min into pre-cooled reaction vessel containing deionized water. Then the mixture was separated by a semi-preparative liquid chromatography column (Waters, C_18_, 300 mm × 7.8 mm, 10 μm). [^18^F]-GA was collected corresponding to the radioactive fraction and evaporated to a residue which was dissolved in sterile phosphate-buffered saline (pH 7.4, 7 mL) and then filtered using Millipore filter (0.22 μm), stored at 4°C (Takano et al., 2011). The process was reproducible and [^18^F]-GA could be duplicable fabricated on the Modular-Lab synthesizers to conform to the required quality control specifications. The stability studies of products were performed by testing the radiochemical purity and pH of aliquots from the validation batches 2, 4, and 6 h after the end of synthesis (Constantinescu et al., 2013b).

### Radiopharmaceutical Quality Control

Radiochemical identity and purity of [^18^F]-GA were determined by ion chromatography (930 Compact IC Flex; Waters) with in-line conduct metric and g-detectors. The column was Shodex IC I-524A (4.6 mm × 100 mm) and the mobile phase was 2.5 mM phthalic acid (pH 4.0). The flow rate was 1.0 mL/min, and the column temperature was kept at 40°C (O’Doherty et al., 2017). The concentration of [^19/18^F]-GA was determined by the calibration curve. Silica gel (silica GF_254_, 5 × 10 cm; Qingdao Hwalong Chemical Co., Ltd.) was selected as a stationary phase and methanol as a mobile phase for radio TLC. The Plate was scanned using a radio-TLC scanner. The radiochemical purity of the product was determined with the [^18^F]-GA peak (Rf = 0.78) as a percentage of total chromatogram radioactivity. The bacterial endotoxin test was performed by Endosafe Portable Test System (Charles River) with a sensitivity of 0.05–5.0 EU/mL.

### [^18^F]-GA Sample Preparation for Activity Assay *in vitro*

The separation process was repeated five times for use in the activity tests. [^18^F]-GA was collected into a plate and evaporated to dryness in a vacuum drying oven at 40°C. After 48 h, radioactive elements had decayed, the residues were stored at 4°C for anti-inflammatory activity tests (Dong et al., 2013; Qingxin et al., 2016).

### Antiinflammatory Activity Tests *in vitro*

HEK 293 T cells were cultured in standard Dulbecco’s modified Eagle’s medium (DMEM; Hyclone) supplemented with 10% fetal bovine serum (FBS; Biological Industries). The cells were placed in 96-well plates at 37°C in an air-CO_2_ environment (95/5% v/v). Cells were grown to 80–90% confluence and incubated for 12 h in DMEM without FBS. Transfection (20 h) was performed according to the manufacturer’s instructions. HEK 293 T cells were pretreated with drugs 1 h prior to TNF-stimulation (5 ng/mL) for 6 h. Dex (10^-5^mol/L) was used as a positive control for NF-κB inhibition. The NF-κB activity was assayed using dual luciferase reporter assay system (Promega) according to the manufacturer’s instructions (Qingxin et al., 2016). Antiinflammatory activity was calculated as the ratio of firefly luciferase activity to Renilla luciferase activity (internal control).

### Animal Grouping and Administration

The mice were grouped by weight and housed in cages of the climate control room (25°C), with a photoperiod 12:12-h. During feeding, mice were free to access food and water. Mice were fasted for 24 h before the experiment. The mice were anesthetized with 4% isoflurane while preparing for scanning (Constantinescu et al., 2013b; Cui et al., 2017). Eighteen healthy male Kunming mice, weighing 20 ± 2 g, were divided into three groups, which were control group, once-administered (OA) group and long-term administration (LA) group. In the OA group, 1 h before the administration of [^18^F]-GA, the mice were orally treated with PG polysaccharides. In the LA group, the mice were orally given the PG polysaccharides for 7 days, but not before the administration of [^18^F]-GA.

### MicroPET Scanning

Rice received a tail vein injection (8.67 ± 2.14 MBq/μg) of [^18^F]-GA and placed in the scanner bed and mouse-imaging room immediately (Constantinescu et al., 2013b; O’Doherty et al., 2017). Firstly, 2 h PET scanning was performed using an Inveon-dedicated PET scanner. The average delay between injection and starting of PET scanning was 3.5 min. Rice were maintained stable under 4% isoflurane anesthesia throughout scanning. The PET list-mode data were dynamically classified into 23 3D sinograms of span and three ring difference. After Fourier recombination, the data were reconstructed using a 2D ordered-subset expectation maximization algorithm. Random events were excluded before reconstruction. The response of detector is normalized using a component-based approach. All dynamic images were corrected for radioactive decay automatically. The PET images were reconstructed by a cone beam algorithm into 480 × 480 × 632 image arrays. After the reconstruction, images were spatially transformed to match the PET images (Constantinescu et al., 2013a,b).

### Image Analysis

The reconstruction of the image was performed using the PMOD software (PMOD Technologies). The 3D volumes of interest (VOIs) were drawn manually of the following organs: brain, heart, liver, lungs, kidneys, cortical bone, and gallbladder. The organs were carefully identified and VOIs were drawn as irregular contours on high-resolution PET images obtained from each mouse (Constantinescu et al., 2013b; Garg et al., 2017). The size of each VOI was less than the actual size of each organ to minimize the effects of PET on adjacent organ spills. The VOIs drawing was verified by a second operator. The VOIs of cortical bone were generated by first using PET image to depict the entire femur bone (Constantinescu et al., 2013b). The VOI was transferred to a dynamic PET image and the mean time-activity curve (TAC) of the decay correction was extracted for each target organ (Constantinescu et al., 2013b).

## Results

### Structural Identification of H-GA and T-GA

Structural identification of the intermediates was achieved by interpreting the molecular ion peaks and ion fragments in the MS/MS spectra and their structures were further characterized by NMR. The purity and identity of the compounds were determined by ^1^H NMR and ^13^C NMR.

### Demonstration of the Bioactivity of NF-κB Inhibitor of [^18^F]-GA

**FIGURE 1:**
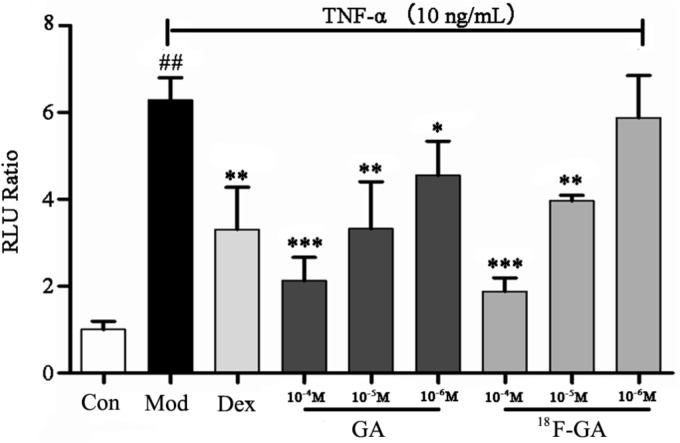
Confirmation of NF-κB inhibition activity of [^18^F]-GA by the luciferase reporter assay system. Each bar represents the mean ± SEM, *n* = 6 per group. ^∗^*p* < 0.05 vs. model group; ^∗∗^*p* < 0.01 vs. model group; ^∗∗∗^*p* < 0.005 vs. model group; ^##^*p* < 0.01 vs. control group.

After the radioactive elements had decayed, [^18^F]-GA was tested for inhibition of NF-κB. GA and [^18^F]-GA (100 μmol/L) were added to HEK 293T which has been stimulated by TNF-α. The activities were evaluated using the luciferase reporter assay system (Qingxin et al., 2016). Dex (10 μmol/L) and GA showed significant NF-κB inhibitory effects (*P* < 0.05), as shown in **Figure 1**. The high concentration and the medium concentration of [^18^F]-GA also showed significant inhibition of NF-κB activity. At the low concentration, it also had a weak anti-inflammatory activity. This showed that the modification of radioactive labeling could maintain its anti-inflammatory activity of GA. [^18^F]-GA was still an effective anti-inflammatory drug, so it can be used as an approximate equivalent drug to investigate the anti-inflammatory activity and distribution of GA.

### Safety Evaluation

In all batches, the radiochemical purity of [^18^F]-GA was greater than 98.6% and the radionuclidic purity was 100%. All batches were sterile and pyrogen free and no impurities were found. The mean and SD of the administered quality of [^19/18^F]-GA was 11 ± 5 mg, and the specific activity was 8.67 ± 2.14 MBq/μg at the time of injection. During the experiment, there was no change in vital signs or electrocardiograms significantly.

### Biodistribution of [^18^F]-GA

**FIGURE 2:**
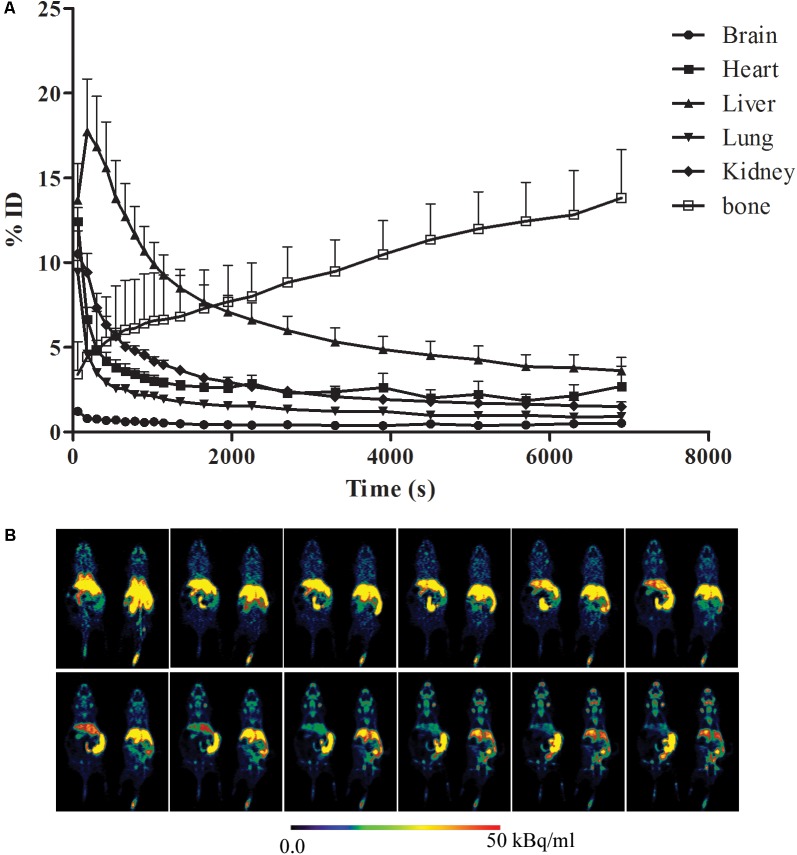
Biodistribution of [^18^F]-GA. **(A)** Organ uptake (expressed as percentage of the injected dose) for [^18^F]-GA over time. The vertical bars represent the standard deviation. **(B)** Whole body biodistribution of [^18^F]-GA over time.

A typical maximum image of biodistribution of the radiotracer from the first (3 min) to the last MicroPET scan (130 min) in the mice was shown in **Figure 2**. After injection of [^18^F]-GA, heart, lung, and kidney can immediately reached a peak, and then quickly declined; the liver reached the peak in 7 min, and then began to gradually decline; the gallbladder peaked at 30 min and maintained on the peak; the free fluoride ion in bone was gradually rising; mice brain concentrations were consistently low because GA could not pass the blood brain barrier. The highest uptake was found in the liver of all rice. In addition, the kidneys can be depicted in the early scan, and the gall bladder becomes visible in subsequent scan. And the heart and lung can be seen all the time. No other organs with an uptake were ingested lower than background (Herzog et al., 2008). As is shown in **Figure 2A**, the uptake data of the mean values of the six mice was listed. To better represent the uptake data of different organs, a typical biodistribution of a mouse is shown in **Figure 2B**. During the time of PET scanning, the radiotracer was found in the gallbladder. Radioactivity was excreted via the biliary. Comparing with the mean values of the single organs in **Table 1**, it is similar to a typical biodistribution of GA that was reported. Therefore, the analysis of the radioactivity could be used to the biodistribution of GA (Mizrahi et al., 2013).

**Table 1:** Organ dose as calculated by OLINDA/EXM 1.1.

Time(s)	Organs (%ID/g)					
	
	Heart	Liver	Lung	Kidney	Brain	Gallbladder	Bone
60	12.13	13.03	8.86	9.67	1.19	11.19	2.98
180	6.52	17.00	4.36	8.98	0.79	14.59	4.00
300	4.75	16.13	3.29	7.13	0.73	19.88	4.38
420	4.07	14.94	2.78	6.26	0.66	23.15	4.84
540	3.71	13.31	2.42	5.59	0.67	24.46	5.17
660	3.50	12.33	2.45	5.00	0.58	26.93	5.45
780	3.33	11.32	2.10	4.73	0.61	29.01	5.52
900	3.17	10.43	2.15	4.52	0.54	30.51	5.77
1020	3.03	9.66	2.09	4.23	0.56	33.20	5.96
1140	2.92	9.05	1.90	4.04	0.52	34.49	6.01
1350	2.78	8.34	1.75	3.71	0.49	36.96	6.16
1650	2.67	7.55	1.66	3.30	0.41	40.92	6.60
1950	2.64	6.96	1.55	3.07	0.42	42.68	6.94
2250	2.82	6.50	1.51	2.79	0.39	44.48	7.22
2700	2.37	5.90	1.34	2.52	0.42	43.79	7.98
3300	2.43	5.27	1.26	2.22	0.37	45.86	8.56
3900	2.63	4.83	1.23	2.06	0.37	46.86	9.42
4500	2.11	4.51	1.05	1.92	0.45	41.98	10.19
5100	2.28	4.27	1.04	1.82	0.38	39.31	10.75
5700	1.96	3.90	1.02	1.75	0.41	36.96	11.15
6300	2.18	3.82	0.97	1.65	0.46	39.06	11.53
6900	2.64	3.64	0.94	1.59	0.48	41.97	12.37


### Changes of Tissue Distribution After Intervention

**FIGURE 3:**
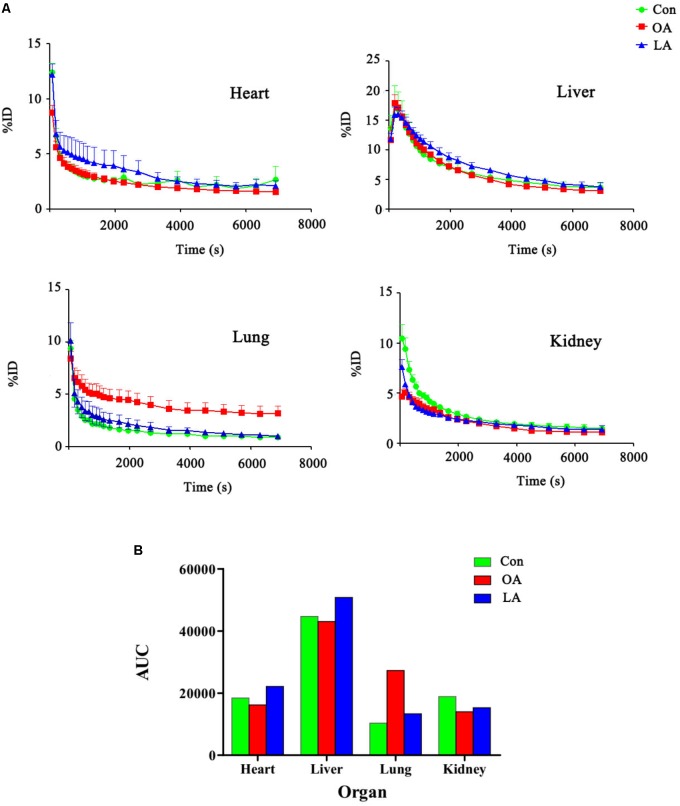
**(A)** The blood drug concentration diagram of organs before and after the intervention of Radix Platycodi. **(B)** The comparison diagram of area under curve of different organs.

**FIGURE 4:**
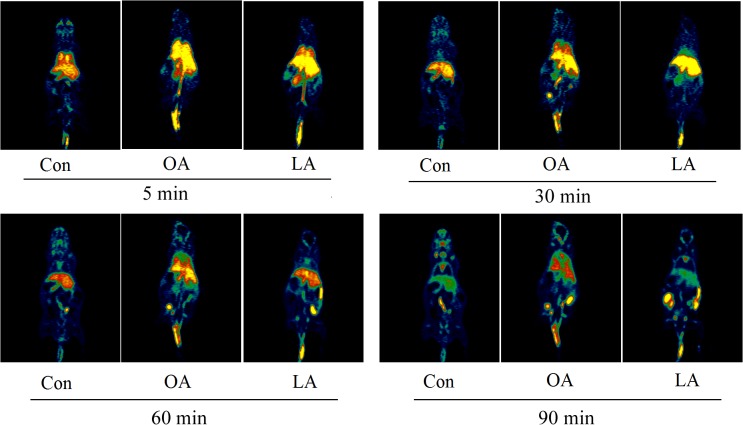
The comparative analysis for the dose of [^18^F]-GA with PG by different mode of administration.

The PG polysaccharides intervention group were scanned by MicroPET, the intensity of radioactivity were recorded and mapped using Prism GraphPad 5. As is shown in **Figure 3A**, there was not obvious intensity change in the heart, liver, and kidney of all groups. However, the intensity of lung in OA group was significantly higher than LA group and control group. Then, after integrating the area under the curve of each organ, the comparison was carried out as shown in **Figure 3B**. In the control group, the [^18^F]-GA concentration of liver was highest, and the lung was lowest. However, after PG polysaccharides intervention, the [^18^F]-GA concentration of lung in the OA group could reach 2.7 times of the control mice, and 2 times of the LA group. Interestingly, there was no significant change in other organs. Comparing the mean values of lungs in **Table 2**, OA group began to show significant differences from the first 7 min. As shown in **Figure 4**, observations at different time intervals also indicated that the [^18^F]-GA concentration of lung in the OA group was higher than in the other groups. It demonstrated that PG polysaccharides can alter the distribution of GA in the lungs, and this change may be related to the mode of administration.

**Table 2:** The %ID/g of [^18^F]-GA in the lungs and the statistical analysis results after PG polysaccharides intervene.

Time(s)	Con	OA	LA	*P*-value		
				
				OA vs. Con	LA vs. Con	OA vs. LA
60	8.86	8.21	8.74	0.528	0.944	0.757
180	4.36	5.95	4.38	0.162	0.988	0.348
300	3.29	5.53	3.63	0.083	0.712	0.281
420	2.78	5.20	3.15	0.048	0.648	0.213
540	2.42	4.85	2.85	0.040	0.577	0.205
660	2.45	4.63	2.90	0.043	0.516	0.228
780	2.10	4.54	2.51	0.025	0.557	0.162
900	2.15	4.53	2.49	0.030	0.582	0.158
1020	2.09	4.35	2.35	0.036	0.675	0.160
1140	1.90	4.25	2.19	0.023	0.590	0.124
1350	1.75	4.17	2.16	0.018	0.418	0.123
1650	1.66	4.06	2.06	0.021	0.406	0.129
1950	1.55	3.95	1.89	0.022	0.393	0.118
2250	1.51	3.79	1.78	0.022	0.456	0.107
2700	1.34	3.54	1.70	0.022	0.257	0.119
3300	1.26	3.17	1.48	0.036	0.361	0.130
3900	1.23	3.06	1.33	0.036	0.719	0.107
4500	1.05	3.07	1.24	0.020	0.382	0.080
5100	1.04	2.96	1.18	0.022	0.440	0.079
5700	1.02	2.88	1.06	0.024	0.815	0.069
6300	0.97	2.95	1.11	0.010	0.402	0.044
6900	0.94	2.85	1.05	0.017	0.556	0.060


## Discussion

### Accuracy of Drug-Time Curves Based on MicroPET

The key to succeed in the distribution and metabolism of natural products by PET is whether the drug curve of PET reflects the data obtained by monitoring our plasma concentration. From the results obtained from PET, the concentration of GA in the brain of the mouse was very low, almost undetectable; indicating that GA could not pass through the blood-brain barrier. GA distributed in the heart, liver, lung, kidney, gallbladder, and other major organs. In addition to the liver and gallbladder, the concentration of other organs was rapidly decreasing. The liver had a clear ascending curve and began to decrease after reaching the peak; the concentration of the gallbladder was always elevated. This showed that the metabolism of GA was mainly through the liver, excretion was mainly through the bile, which was consistent with the literature reported (Gao et al., 2004). From the concentration–time curve, it can be found that the pharmacokinetic profile of GA was similar in the absorption phase. The T_max_, C_max_ value didn’t show significant difference (Taira et al., 2004). GA is metabolized by the liver and excreted by bile (Ploeger et al., 2001). The similarity of pharmacokinetic profile of GA in the distribution phase showed that the established PET technique is consistent with the data of traditional plasma concentration monitoring and could be used for online real-time detection of plasma concentrations.

### Study on the Function of GA and PG Polysaccharides

In the theory of TCM, the combination of licorice and *Platycodon grandiflorum* has been widely used (Yang et al., 2017), but its specific mechanism of action has yet to be further clarified. Previous study has been designed to investigate the metabolism of GA using human liver microsomes (HLM) and recombinant cytochrome P450 (CYP) isoforms *in vitro*. The results indicated that GA was mainly metabolized by CYP3A4 (Zhang et al., 2017a). However, the effect of a single oral administration of GA on rat pharmacokinetics was limited, suggesting that GA can merely act as a weak inhibitor of CYP3A-mediated drug metabolism *in vivo* (Kim et al., 2015). Moreover, GA was mentioned to be Central Nervous System (CNS) restricted as well as biliary excreted suggests GA may be substrate of the efflux transporters mentioned (Chen et al., 2017; Zhang et al., 2017b). Mechanistically, PG polysaccharides treatment was found significantly inhibit the transduction of NF-κB signal pathway (Vander Broek et al., 2014; Zhang et al., 2017b), therefore activity assay confirmed that NF-κB-directed transcription was attenuated in the presence of PG polysaccharides (Chang et al., 2015; Zhang et al., 2017b). Consistently, MMP-2 and MMP-9 as the downstream target genes of NF-κB (Dionato et al., 2012), were down-regulated by PG polysaccharides. These results indicated that PG polysaccharide may exerts its biological effects by inactivating NF-κB mediated transcriptional activity. We provided additional evidence that it is speculated that PG polysaccharides may affect the process of other drugs *in vivo*, as well as the distribution of drug components *in vivo*. In this study, we examined the radioactive labeled GA from mice by MicroPET, and evaluated the therapeutic effect of two kinds of administration of PG polysaccharides before injection. The results showed that the combination of GA and PG polysaccharides could effectively change the distribution of drugs.

## Conclusion

Our data demonstrated that MicroPET could be used to study the biodistribution of natural products. The combination of GA and PG polysaccharides could effectively change the distribution of drugs, which was largely associated with inhibition of NF-κB activity. Administration of GA and PG polysaccharides at the same time was able to play a synergistic anti-inflammatory effect. These findings suggested that PG polysaccharides may have the potential to treat or assit in the treatment of pneumonia. Thus, MicroPET imaging combined with the classical pharmacological method is recommended as preliminary clinical trial before they are applied to subsequent clinical investigations. And the modern prescription compatibility research has been intuitively revealed *in vivo*.

## Ethics Statement

This study was carried out in accordance with the recommendations of “Principle of Laboratory Animal Care (NIH Publication No. 85-23, revised 1985) guidelines,” “Animal Ethics Committee of Nankai University.” The protocol was approved by the “Animal Ethics Committee of Nankai University.”

## Author Contributions

GB and QC designed the study. QC performed the experiments, acquired and analyzed the data, and drafted and edited the manuscript. YL, MZ, YH, and CY assisted with experiments. GB contributed to data discussion and reviewed the manuscript.

## Conflict of Interest Statement

The authors declare that the research was conducted in the absence of any commercial or financial relationships that could be construed as a potential conflict of interest.
